# Propofol Sedation for Oesophago-Gastro-Duodenoscopy

**DOI:** 10.1155/DTE.6.37

**Published:** 1999

**Authors:** J. M. Watt, J. W. L. Fielding

**Affiliations:** Queen Elizabeth Hospital Edgbaston Birmingham B15 2TH UK

## Abstract

A questionnaire was sent to 53 patients who had undergone an upper gastrointestinal endoscopy under total intravenous anaesthesia (TIVA) using intermittent Propofol. All of the patients would accept the same technique again. Out of 20 patients who had previously had the procedure performed under Diazepam sedation, 18 preferred the use of Propofol. This technique can only be used with an anaesthetist present.


*Diagnostic and Therapeutic Endoscopy*, Vol. 6, pp. 37–38
Reprints available directly from the publisher
Photocopying permitted by license only

© 1999 OPA (Overseas Publishers Association) N.V.
Published by license under
the Harwood Academic Publishers imprint,
part of The Gordon and Breach Publishing Group.
Printed in Malaysia.

### Propofol Sedation for Oesophago-Gastro-Duodenoscopy

J.M. WATT* and J.W.L. FIELDING

*Queen Elizabeth Hospital, Edgbaston, Birmingham, B15 2TH, UK*

*(Received 30 March 1999; Revised 12 May 1999; In final form 10 June 1999)*

**A questionnaire was sent to 53 patients who had undergone an upper gastrointestinal endoscopy under total intravenous anaesthesia (TIVA) using intermittent Propofol. All of the patients would accept the same technique again. Out of 20 patients who had previously had the procedure performed under Diazepam sedation, 18 preferred the use of Propofol.**

**This technique can only be used with an anaesthetist present.**

*Keywords:* Endoscopy, Propofol, Sedation, TIVA

Fibre-optic gastroscopy is usually performed under sedation most commonly using Diazepam. This procedure is not always satisfactory, the level of sedation is variable: it can be ineffective and result in an unpleasant experience for the patient. Inadequate sedation may result in a poor view of the entire gastrict mucosa. In addition, the recovery period is prolonged.

Propofol could potentially be used for anaesthetising patients undergoing gastroscopy. Because of its narrower therapeutic range, an anaesthetist is required to monitor and maintain the airway [1,2]. This paper evaluates the use of Propofol for anaesthesia during upper gastrointestinal (GI) tract endoscopy.

### METHODS

Fifty three patients underwent upper GI endoscopy using intermittent intravenous Propofol as the sole method of anaesthesia. Propofol was administered by slow intravenous injection until loss of eyelash reflex. The required dose varied from 120 to 300 mg. Incremental doses of 50 mg were injected in response to coughing or purposeful movement by the patient providing that the oxygen saturation remained above 90%. All patients received oxygen through nasal cannulae and the oxygen saturation was monitored using a pulse oximeter. The procedure was interrupted and the patient ventilated with 100% oxygen by facemask if the oxygen saturation

* Corresponding author. Tel.: +44-121-627-2272. Fax: +44-121-627-2273.

fell below 85%. A questionnaire was posted to these 53 patients who were asked to report any side effects, the duration of post-operative drowsiness and whether they would wish to have the same anaesthetic procedure again. In addition, patients who had had a previous gastroscopy under conventional sedation were asked which method they preferred and for what reason. In addition to these, the results of 537 endoscopies done under this method has been audited for complications from both the anaesthesia and the gastroscopy.

### RESULTS

Fifty three questionnaires were sent out and 48 replies received. All the respondents said they would have the same anaesthetic again and only 5 reported side effects:

(1) Change in bowel habit for 2 weeks;
(2) Dizzy for 2 days;
(3) Headache;
(4) Sore throat and headache;
(5) Sore throat, abdomen and chest sore.

Twenty patients has previously had gastroscopy performed using a different method of sedation. Nineteen were sedated with Diazepam and 1 had no sedation at all. Eighteen expressed a preference for Propofol and 2 said that Diazepam or Propofol was equally acceptable. The main reason for preferring Propofol was that the technique was more comfortable [3] and recovery was more rapid [4]. In addition to these, 537 other patients have received Propofol sedation for gastroscopy. Two of these have required the insertion of a laryngeal mask and assisted ventilation with oxygen. This has been short term and the patients have been discharged as day cases. There have been no complications associated with the endoscopy.

### DISCUSSION

Oesophago-gastro-duodenoscopy is the main method of investigating patients with upper gastrointestinal symptoms. This is a safe procedure, the complication rate reported from the technical part of the gastroscopy is 0.06–0.5 [3]. The potential risk is usually related to the sedation and the Diazepam has a reported mortality of 1 : 2000 [4]. Most endoscopies are done as day cases without the assistance of an anaesthetist. This technique with Propofol reported in this paper certainly requires the presence of an anaesthetist. It is more acceptable to the patient and in over 500 endoscopies there has been no significant complication. This method allows a very detailed endoscopy to be undertaken, the stomach can be well distended, without regurgitation of fluid or air, allowing excellent visualisation of the entire gastric mucosa.

### *References*

[1] Newson, C., Joshi, G.P. and White, P.F. Comparison of Propofol administration techniques for sedation during monitored anesthesia care. *Anesthesia and Analgesia* 1995; **81**(3): 486–91.
[2] Lechner, J. and Haberstroh, J. Propofol: a short term narcotic tested in gastroduodenoscopy in the cat. *Tierarztliche Peraxiz* 1993; **21**(1): 71–3.
[3] Froehlich, F., Governs, J.J. and Fried, M. Conscious sedation, clinically relevant complication and monitoring of endoscopy: results or a nation-wide survey in Switzerland. *Endoscopy* 1994; **26**(2): 231–4.
[4] Quine, M.A. *et al.* Prospective audit of upper gastrointestinal endoscopy in two regions of England: safety, staffing and sedative methods. *Gut* 1996; **36**(3): 462–7.

